# Synthesis, crystal structure and Hirshfeld surface analysis of 1-ferrocenylundecane-1,11-diol

**DOI:** 10.1107/S205698902101358X

**Published:** 2022-01-07

**Authors:** C. John McAdam, Jim Simpson

**Affiliations:** a Department of Chemistry, University of Otago, PO Box 56, Dunedin, New Zealand

**Keywords:** crystal structure, ferrocene, undecane-1,11-diol, hydrogen bonds, C—H⋯π contact, Hirshfeld surface analysis

## Abstract

The racemic title compound, [Fe(C_5_H_5_)(C_16_H_27_O_2_)], comprises an α,ω-diol-substituted undecyl chain with a ferrocenyl substituent at at one terminus. The alkane chain is inclined to the substituted ring of the ferrocene grouping by 84.22 (13)°. The ferrocene rings are almost eclipsed and parallel. The crystal structure features O—H⋯O and C—H⋯O hydrogen bonds and C—H⋯π contacts that stack the mol­ecules along the *c-*axis direction. A Hirshfeld surface analysis reveals that H⋯H inter­actions (83.2%) dominate the surface contacts.

## Abstract 2

The mol­ecular and crystal structure of a ferrocenyl derivative with an undecyl-1,11-diol chain on one cyclo­penta­dienyl ring is reported: O—H⋯O, C—H⋯O and C—H⋯π(ring) contacts occur in the extended structure.

## Chemical context

The title compound, **1**, is a rare example of a ferrocene mol­ecule substituted with an extended, in this instance 11-membered, alkane chain. It was synthesized to provide a ferrocenyl-substituted diol for the preparation of polyesters with regular pendant electroactive groups. Similar ferrocenyl *neo*-pentyl diol-derived terephthalate polymers have been shown to display inter­esting electrochemical properties (McAdam *et al.*, 2008*a* ▸,*b* ▸). Friedel–Crafts methodology (Saji *et al.*, 1991 ▸) provided the 1-ferrocenyl-undec-10-en-1-one precursor. This was reduced to the racemic alcohol 1-ferrocenyl-undec-10-en-1-ol (**2**) using LiAlH_4_. Enanti­omeric selection of the individual chiral forms should be possible using more complex synthetic methodology (Ursini *et al.*, 2006 ▸; Schwink *et al.*, 1998 ▸), but was deemed unnecessary for our purposes. Hydro­boration of ferrocenylalkenes has been previously reported (Lo Sterzo *et al.*, 1984 ▸) using borane generated *in situ* from NaBH_4_/BF_3_·OEt_2_. Predictably, this method was unsuitable as a means of preparing **1** from **2**, the ferrocenyl­methanol moiety being susceptible to attack by BF_3_, and the resultant loss of OH^−^ abetted by the formation of the stable α-ferrocenyl carbenium ion. This prediction was borne out by experiment, the Lewis acid attack resulting in synthesis of 1-ferrocenyl-undec-10-ene and 1-ferrocenyl-undec-11-ol. Instead, a successful synthesis of **1** was achieved using hydro­boration of **2** with 9-BBN. 
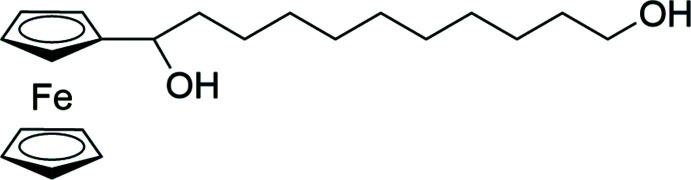


## Structural commentary

**Figure 1:**
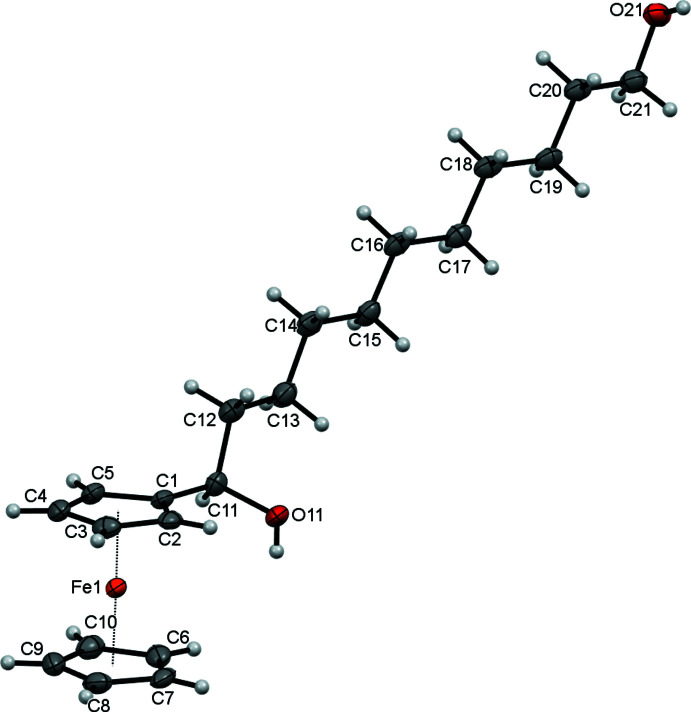
The mol­ecular structure of **1** with ellipsoids drawn at the 50% probability level.

The title compound, [Fe(C_5_H_5_)(C_16_H_27_O_2_)], comprises a ferrocene unit that carries a well-ordered undecane chain (atoms C11–C21) with hydroxyl substituents at the 1 and 11 positions along the chain (Fig. 1 ▸). The C13—C12—C11—O11 and C19—C20—C21—O21 torsion angles are 60.9 (3) and 173.9 (2)°, respectively. Atom C11 is a stereogenic centre: in the arbitrarily chosen asymmetric mol­ecule it has an *R* configuration, but crystal symmetry generates a racemic mixture. The alkane chain is almost planar with the r.m.s. deviation from the best fit plane through all 11 C atoms being 0.129 Å. This plane is nearly orthogonal to the substituted ferrocene ring with an angle of 84.22 (13)° between them. The C_11_ undecyl chain in **1** is conformationally extended with the typical anti­periplanar (Kane & Hersh, 2000 ▸) arrangement for C_*n*_–C_*n*+3_ groupings and a C11⋯C21 separation of 12.627 (4) Å. The C1–C5 and C6–C10 cyclo­penta­dienyl rings of the ferrocenyl group are approximately 3° from being eclipsed and are almost coplanar with a dihedral angle of 1.7 (2)° between them; the separation of the ring centroids is is 3.298 (2) Å.

## Supra­molecular features

**Figure 2:**
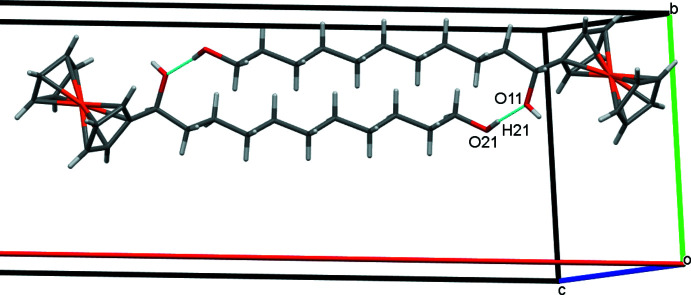
Inversion dimers of **1** in the *ab* plane with O—H⋯O hydrogen bonds shown as blue lines.

**Figure 3:**
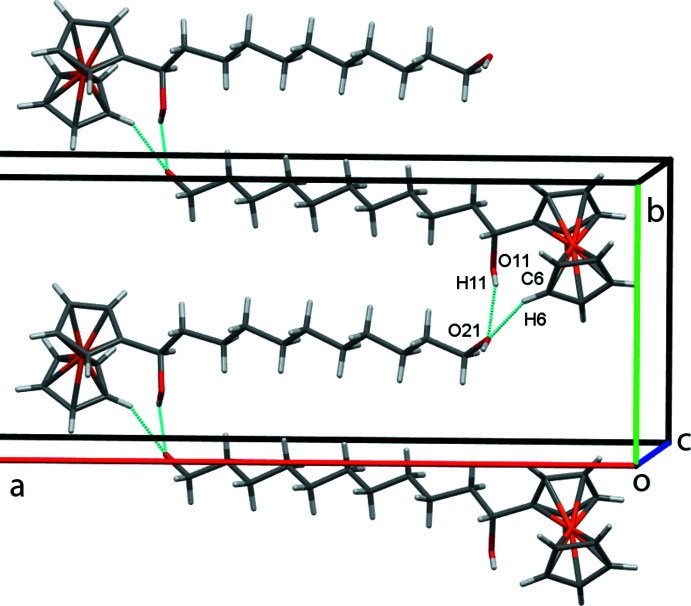
Chains of mol­ecules of **1** propagating along *b* with O—H⋯O and C—H⋯O hydrogen bonds shown as blue lines.

**Figure 4:**
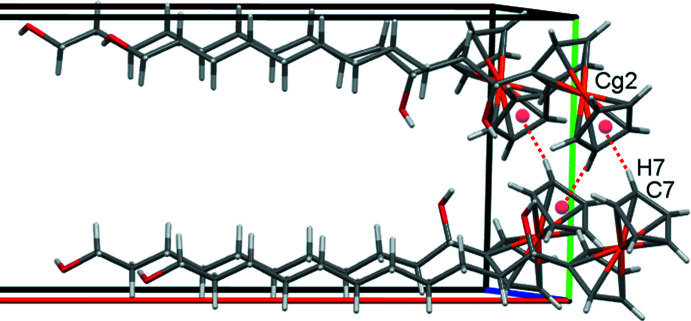
Double chains of mol­ecules of **1** along *c. *Cg** 2 is the centroid of the C6–C10 cyclo­penta­dienyl ring, shown here as red spheres, with the C—H⋯π contacts drawn as dashed red lines.

**Figure 5:**
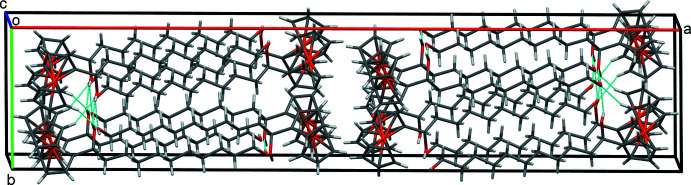
Overall packing of **1** viewed along the *c*-axis direction.

In the crystal of **1**, inversion dimers form in the *ab* plane through pairwise classical O21—H21⋯O11 hydrogen bonds (Table 1 ▸), which generate 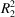 (28) ring motifs (Fig. 2 ▸). Additional classical O11—H11⋯O21 hydrogen bonds, supported by weaker non-classical C6—H6⋯O21 contacts, form alternating chains of mol­ecules along the *b-*axis direction and O21 acts as a double acceptor (Fig. 3 ▸). A weak C7—H7⋯*Cg*2 (H⋯π = 2.89 Å, C—H⋯π = 164°) contact involving the unsubstituted ring of the ferrocene unit forms double chains of mol­ecules propagating along the *c*-axis direction (Fig. 4 ▸) where *Cg*2 is the centroid of the C6–C10 cyclo­penta­dienyl ring. Overall these various contacts combine to stack the mol­ecules of **1** along the *c-*axis direction in two discrete, parallel and well-separated columns (Fig. 5 ▸).

## Hirshfeld surface analysis

**Figure 6:**
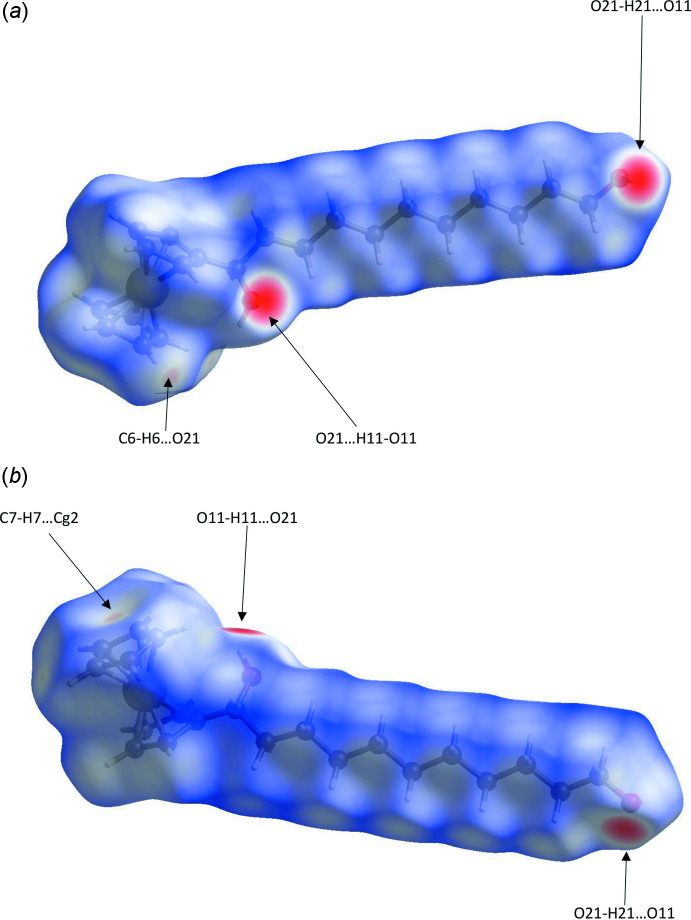
Hirshfeld surfaces for opposite faces of **1** mapped over *d*_norm_ in the range −0.67 to 1.35 a.u.

**Figure 7:**
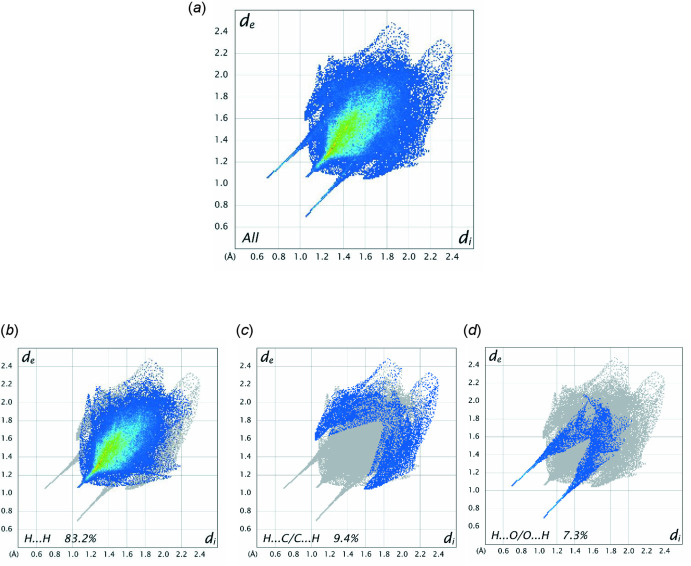
A full two-dimensional fingerprint plot for **1**, (*a*), together with (*b*)–(*d*) separate principal contact types for the mol­ecule: H⋯H, H⋯C/C⋯H and H⋯O/O⋯H, respectively.

Further details of the inter­molecular inter­actions in **1** were obtained using Hirshfeld surface analysis (Spackman & Jayatilaka, 2009 ▸) with Hirshfeld surfaces and two-dimensional fingerprint plots generated with *Crystal Explorer* (Turner *et al.*, 2017 ▸). Hirshfeld surfaces for opposite faces of **1** are shown in Fig. 6 ▸(*a*) and (*b*). Bold red areas on the Hirshfeld surfaces correspond to the classical O—H⋯O hydrogen bonds while the weaker C—H⋯O and C—H⋯π contacts appear as faint red circles. Fingerprint plots (Fig. 7 ▸) reveal that H⋯H inter­actions dominate the surface contacts, as would be expected for a mol­ecule with such a predominance of H atoms, with H⋯C/C⋯H and H⋯O/O⋯H contacts also making significant contributions to the surface (Table 2 ▸).

## Database survey

Ferrocene derivatives with pendant C_*n*_ alkyl chains (*n* ≥ 11) are uncommon and the majority of such structures that appear in the Cambridge Structural Database (version 5.41 Nov 2019 with updates to March 2020; Groom *et al.*, 2016 ▸) are bis-ferrocenyl complexes. These include 1,12-bis-ferrocenyldo­decane (refcodes FOHHAM and FOHHAM01; Bequeath *et al.*, 2005 ▸, Wedeking *et al.*, 2006*a* ▸) and the tetra­decane, octa­decane and docosane derivatives (VEFXIO, VEFXOU, VEFXUA; Wedeking *et al.*, 2006*a* ▸). *n*-Tetra­decyl­ferrocene (MEFRUL; Wedeking *et al.*, 2006*b* ▸) is the only mono-ferrocene with an unsubstituted alkane chain, while our earlier report of the structure of 11-bromo-1-ferrocenylundecan-1-one (LICNIV; McAdam *et al.*, 2007 ▸) is the sole example of such a structure with substitution on the alkane chain. Inter­estingly, the structure of the related 1,11-undeca­nediol (HIYHAY; Nakamura *et al.*, 1999 ▸) has also been reported. However, α,ω-di­hydroxy­alkane (C_*n*_, *n* ≥ 10) structures are uncommon and often crystallize as co-crystals, see, for example, KEXZOD and KEXZUJ (Loehlin *et al.*, 2007 ▸) OTIZEX, OTIZIB, OTIZOH and OTIZUN (Martí-Rujas *et al.*, 2011 ▸).

## Synthesis and crystallization

**Figure 8:**
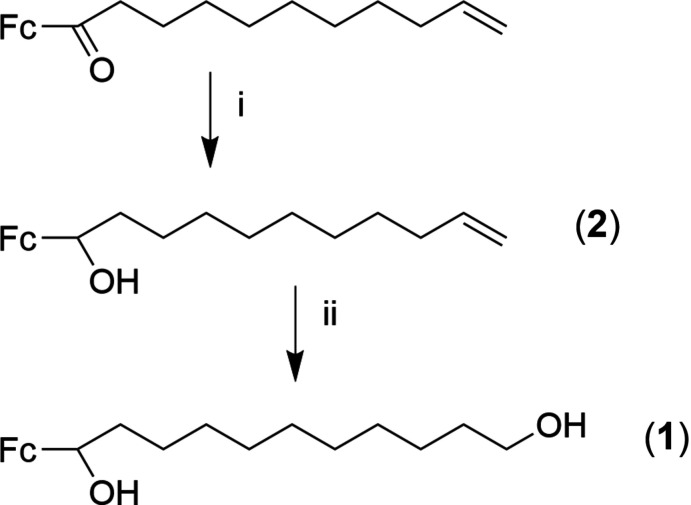
Preparation scheme for **1**; (i) LiAlH_4_, Et_2_O; (ii) 9-BBN, THF.

The title compound **1** was prepared in two steps from 1-ferrocenyl-undec-10-en-1-one (Evans *et al.*, 2008 ▸) *via* a lithium aluminium hydride reduction followed by hydro­boration with 9-borabi­cyclo­[3.3.1]nonane (9-BBN) (Aristoff *et al.*, 1985 ▸), Fig. 8 ▸. LiAlH_4_ (0.10 g, 2.6 mmol) was added to 1-ferrocenyl-undec-10-en-1-one (0.615 g, 1.75 mmol) in Et_2_O (10 mL) at 273 K and stirred for 1 h before quenching with a few drops of water. The ether fraction was rinsed with saturated NaCl solution and dried over MgSO_4_. The solvent was removed under vacuum to give 0.61 g (99%) of the yellow oil 1-ferrocenyl-undec-10-en-1-ol. To this oil, without further purification, in THF (10 ml) was added a solution of 9-BBN (0.5 *M* in hexane, 3.5 mmol), the mixture stirred at room temperature for 18 h before quenching with a few drops of water. The pH was raised to 8.5 with NaOH, then hydrogen peroxide (30% in H_2_O, 7 ml) was added and the mixture allowed to stir for another 2 h. The organic layer was rinsed with saturated NaCl solution and dried over MgSO_4_. Column chromatography on SiO_2_ with CH_2_Cl_2_ eluted a trace of the unreacted alcohol. Further elution with EtOAc/CH_2_Cl_2_ gave the title compound **1** as a yellow solid (0.60 g, 94%). X-ray quality crystals were grown from the mixed solvents of CH_2_Cl_2_ layered with hexane. Analysis calculated for C_21_H_32_O_2_Fe: C, 67.74; H, 8.66. Found: C, 67.94; H, 8.92%. ^1^H NMR (CDCl_3_): 4.30 (*m*, 1H, –C*H*OH–), 4.24 (*m*, 1H, C_5_ *H*_4_), 4.20 (*s*, 5H, Cp), 4.17 (*m*, 3H, C_5_ *H*_4_), 3.64 (*m*, 2H, –C*H*_2_—OH), 1.92 [*d* (*J* = 4 Hz), 1H, Fc-CHO*H*], 1.7–1.3 [*m*, 18H, –(C*H*_2_)_9_–]. ^13^C NMR (CDCl_3_): 94.7 (Fc *ipso*), 69.7 (–*C*HOH–), 68.3 (Cp), 67.9, 67.7, 67.3, 65.2 (Fc—*C*α & β), 63.2 (–*C*H_2_OH), 38.3, 32.9, 29.6, 29.6, 29.5, 29.5, 26.1, 25.8 (–*C*H_2_–). UV–vis (CH_2_Cl_2_): 325 (*90*), 440 (*110*) nm (*ɛ*).

## Refinement

Crystal data, data collection and structure refinement details are summarized in Table 3 ▸. The O-bound H atoms were located in a difference-Fourier map and their coordinates refined with U_iso_(H) = 1.5 U_eq_(O). All H-atoms bound to C were refined using a riding model with C—H = 0.95–1.00 Å and *U*_iso_(H) = 1.2*U*_eq_(C). Despite repeated attempts to grow crystals of better quality, the crystals obtained were weakly diffracting and the extent of diffraction observed is poor with sin (θ_max_)/λ = 0.544 (2θ_max_ = 44.5°). Despite this, the structure solved and refined adequately.

## Supplementary Material

Crystal structure: contains datablock(s) I. DOI: 10.1107/S205698902101358X/hb8007sup1.cif

Structure factors: contains datablock(s) I. DOI: 10.1107/S205698902101358X/hb8007Isup2.hkl

CCDC reference: 2130725

Additional supporting information:  crystallographic information; 3D view; checkCIF report

## Figures and Tables

**Table 1:** Hydrogen-bond geometry (Å, °)

*D*—H⋯*A*	*D*—H	H⋯*A*	*D*⋯*A*	*D*—H⋯*A*
O11—H11⋯O21^i^	0.76	2.06	2.755 (3)	152
O21—H21⋯O11^ii^	0.83	1.90	2.726 (3)	175
C6—H6⋯O21^i^	0.95	2.60	3.380 (4)	140

Symmetry codes: (i) -x+{\script{1\over 2}}, y-{\script{1\over 2}}, -z+{\script{3\over 2}}; (ii) -x+{\script{1\over 2}}, -y+{\script{3\over 2}}, -z+2.

**Table 2:** Percentage contributions to the Hirshfeld surface of **1**

Contents	Included surface area
H⋯H	83.2
H⋯C/C⋯H	9.4
H⋯O/O⋯H	7.3

**Table 3:** Experimental details

Crystal data	
Chemical formula	[Fe(C_5_H_5_)(C_16_H_27_O_2_]
*M*_r_	372.31
Crystal system, space group	Monoclinic, *C*2/*c*
Temperature (K)	92
*a*, *b*, *c* (Å)	47.641 (3), 10.1522 (7), 7.8747 (6)
β (°)	97.091 (4)
*V* (Å^3^)	3779.6 (5)
*Z*	8
Radiation type	Mo *K*α
μ (mm^−1^)	0.81
Crystal size (mm)	0.32 × 0.14 × 0.04

Data collection	
Diffractometer	CCD area detector
Absorption correction	Multi-scan (*SADABS*; Bruker, 2011 ▸)
*T*_min_, *T*_max_	0.784, 1.000
No. of measured, independent and observed [*I* > 2σ(*I*)] reflections	16666, 2527, 2150
*R*_int_	0.049
θ_max_ (°)	22.7
(sin θ/λ)_max_ (Å^−1^)	0.544

Refinement	
*R*[*F* ^2^ > 2σ(*F* ^2^)], *wR*(*F* ^2^), *S*	0.037, 0.106, 1.06
No. of reflections	2527
No. of parameters	221
H-atom treatment	H atoms treated by a mixture of independent and constrained refinement
Δρ_max_, Δρ_min_ (e Å^−3^)	0.64, −0.31

Computer programs: *APEX2* and *SAINT* (Bruker, 2011 ▸), *SHELXT* (Sheldrick, 2015*a* ▸), *SHELXL2018/1* (Sheldrick, 2015*b* ▸), *TITAN* (Hunter & Simpson, 1999 ▸), *Mercury* (Macrae *et al.*, 2020 ▸), *enCIFer* (Allen *et al.*, 2004 ▸), *PLATON* (Spek, 2020 ▸), *publCIF* (Westrip 2010 ▸) and *WinGX* (Farrugia 2012 ▸).

## supplementary crystallographic information

### Crystal data

**Table:**

[Fe(C_5_H_5_)(C_16_H_27_O_2_]	*F*(000) = 1600
*M**_r_* = 372.31	*D*_x_ = 1.309 Mg m^−^^3^
Monoclinic, *C*2/*c*	Mo *K*α radiation, λ = 0.71073 Å
*a* = 47.641 (3) Å	Cell parameters from 4218 reflections
*b* = 10.1522 (7) Å	θ = 2.4–22.4°
*c* = 7.8747 (6) Å	µ = 0.81 mm^−^^1^
β = 97.091 (4)°	*T* = 92 K
*V* = 3779.6 (5) Å^3^	Plate, yellow
*Z* = 8	0.32 × 0.14 × 0.04 mm

### Data collection

**Table:**

CCD area detector diffractometer	2527 independent reflections
Radiation source: sealed tube	2150 reflections with *I* > 2σ(*I*)
Graphite monochromator	*R*_int_ = 0.049
phi and ω scans	θ_max_ = 22.7°, θ_min_ = 1.7°
Absorption correction: multi-scan (SADABS; Bruker, 2011)	*h* = −51→51
*T*_min_ = 0.784, *T*_max_ = 1.000	*k* = −11→11
16666 measured reflections	*l* = −8→7

### Refinement

**Table:**

Refinement on *F*^2^	0 restraints
Least-squares matrix: full	Hydrogen site location: inferred from neighbouring sites
*R*[*F*^2^ > 2σ(*F*^2^)] = 0.037	H atoms treated by a mixture of independent and constrained refinement
*wR*(*F*^2^) = 0.106	*w* = 1/[σ^2^(*F*_o_^2^) + (0.0649*P*)^2^ + 3.6265*P*] where *P* = (*F*_o_^2^ + 2*F*_c_^2^)/3
*S* = 1.06	(Δ/σ)_max_ = 0.001
2527 reflections	Δρ_max_ = 0.64 e Å^−^^3^
221 parameters	Δρ_min_ = −0.31 e Å^−^^3^

### Special details

**Table:**

Geometry. All esds (except the esd in the dihedral angle between two l.s. planes) are estimated using the full covariance matrix. The cell esds are taken into account individually in the estimation of esds in distances, angles and torsion angles; correlations between esds in cell parameters are only used when they are defined by crystal symmetry. An approximate (isotropic) treatment of cell esds is used for estimating esds involving l.s. planes.
Refinement. A reflection effected by the beamstop and two reflections with Fo >>> Fc were omitted from the final refinement cycles.

### Fractional atomic coordinates and isotropic or equivalent isotropic displacement parameters (Å^2^)

**Table:**

	*x*	*y*	*z*	*U*_iso_*/*U*_eq_	
C1	0.08497 (6)	0.8323 (3)	0.3254 (4)	0.0242 (7)	
C2	0.06289 (6)	0.7864 (3)	0.4173 (4)	0.0249 (7)	
H2	0.064664	0.719397	0.502125	0.030*	
C3	0.03794 (7)	0.8572 (3)	0.3612 (4)	0.0281 (7)	
H3	0.020049	0.845762	0.400751	0.034*	
C4	0.04438 (6)	0.9489 (3)	0.2348 (4)	0.0263 (7)	
H4	0.031523	1.009645	0.175642	0.032*	
C5	0.07318 (6)	0.9340 (3)	0.2122 (4)	0.0253 (7)	
H5	0.083009	0.982862	0.135301	0.030*	
Fe1	0.05212 (2)	0.76105 (4)	0.16036 (5)	0.02107 (19)	
C6	0.06708 (7)	0.6085 (3)	0.0268 (4)	0.0304 (8)	
H6	0.085748	0.573937	0.042214	0.036*	
C7	0.04426 (7)	0.5650 (3)	0.1116 (4)	0.0302 (8)	
H7	0.044913	0.496850	0.194681	0.036*	
C8	0.02022 (7)	0.6416 (3)	0.0500 (4)	0.0331 (8)	
H8	0.001833	0.633157	0.083682	0.040*	
C9	0.02838 (7)	0.7320 (3)	−0.0694 (4)	0.0336 (8)	
H9	0.016408	0.795724	−0.129627	0.040*	
C10	0.05726 (7)	0.7129 (3)	−0.0855 (4)	0.0321 (8)	
H10	0.068119	0.760822	−0.157882	0.039*	
C11	0.11527 (6)	0.7882 (3)	0.3455 (4)	0.0274 (7)	
H11A	0.122055	0.786316	0.230314	0.033*	
O11	0.11807 (5)	0.65849 (18)	0.4193 (3)	0.0298 (5)	
H11	0.1142 (7)	0.607 (2)	0.350 (3)	0.045*	
C12	0.13452 (6)	0.8767 (3)	0.4624 (4)	0.0298 (7)	
H12A	0.128323	0.875560	0.577879	0.036*	
H12B	0.132689	0.968142	0.418890	0.036*	
C13	0.16570 (6)	0.8354 (3)	0.4768 (4)	0.0313 (8)	
H13A	0.166977	0.739640	0.500383	0.038*	
H13B	0.172652	0.850703	0.365045	0.038*	
C14	0.18500 (6)	0.9069 (3)	0.6140 (4)	0.0293 (7)	
H14A	0.176768	0.902415	0.723312	0.035*	
H14B	0.186169	1.000796	0.581914	0.035*	
C15	0.21465 (6)	0.8487 (3)	0.6398 (4)	0.0286 (7)	
H15A	0.213202	0.753762	0.665954	0.034*	
H15B	0.222958	0.856302	0.531020	0.034*	
C16	0.23466 (6)	0.9126 (3)	0.7808 (4)	0.0283 (7)	
H16A	0.226058	0.908646	0.888857	0.034*	
H16B	0.236941	1.006643	0.752146	0.034*	
C17	0.26376 (6)	0.8485 (3)	0.8092 (4)	0.0277 (7)	
H17A	0.261428	0.754213	0.836426	0.033*	
H17B	0.272404	0.853228	0.701314	0.033*	
C18	0.28391 (6)	0.9108 (3)	0.9514 (4)	0.0283 (7)	
H18A	0.286265	1.005048	0.924393	0.034*	
H18B	0.275331	0.905800	1.059485	0.034*	
C19	0.31288 (6)	0.8462 (3)	0.9785 (4)	0.0286 (7)	
H19A	0.310578	0.752371	1.007805	0.034*	
H19B	0.321323	0.849641	0.869788	0.034*	
C20	0.33312 (6)	0.9104 (3)	1.1183 (4)	0.0275 (7)	
H20A	0.334116	1.005977	1.094737	0.033*	
H20B	0.325615	0.899498	1.229180	0.033*	
C21	0.36243 (6)	0.8533 (3)	1.1321 (4)	0.0307 (8)	
H21A	0.361643	0.759959	1.167703	0.037*	
H21B	0.369024	0.855208	1.017765	0.037*	
O21	0.38252 (4)	0.9210 (2)	1.2505 (3)	0.0337 (6)	
H21	0.3814 (5)	0.894 (3)	1.349 (4)	0.051*	

### Atomic displacement parameters (Å^2^)

**Table:**

	*U*^11^	*U*^22^	*U*^33^	*U*^12^	*U*^13^	*U*^23^
C1	0.0313 (17)	0.0143 (15)	0.0256 (17)	−0.0044 (12)	−0.0020 (13)	−0.0012 (13)
C2	0.0363 (18)	0.0187 (15)	0.0187 (16)	−0.0042 (13)	−0.0007 (13)	−0.0049 (13)
C3	0.0297 (17)	0.0256 (17)	0.0296 (17)	−0.0016 (13)	0.0061 (14)	−0.0079 (14)
C4	0.0295 (17)	0.0178 (16)	0.0300 (17)	0.0025 (12)	−0.0024 (13)	−0.0033 (13)
C5	0.0306 (17)	0.0154 (15)	0.0290 (17)	−0.0028 (12)	0.0000 (13)	0.0001 (13)
Fe1	0.0263 (3)	0.0149 (3)	0.0213 (3)	−0.00056 (17)	−0.00010 (19)	−0.00066 (17)
C6	0.0358 (19)	0.0259 (17)	0.0280 (18)	0.0068 (14)	−0.0018 (14)	−0.0080 (14)
C7	0.048 (2)	0.0149 (15)	0.0263 (17)	−0.0020 (14)	0.0010 (15)	−0.0038 (13)
C8	0.0325 (18)	0.0266 (17)	0.039 (2)	−0.0059 (14)	−0.0010 (15)	−0.0088 (15)
C9	0.042 (2)	0.0236 (17)	0.0314 (19)	0.0036 (14)	−0.0105 (15)	−0.0024 (14)
C10	0.047 (2)	0.0248 (17)	0.0244 (18)	−0.0047 (15)	0.0044 (15)	−0.0020 (14)
C11	0.0305 (17)	0.0197 (16)	0.0306 (18)	−0.0005 (13)	−0.0014 (14)	0.0052 (14)
O11	0.0372 (13)	0.0157 (11)	0.0339 (13)	0.0004 (9)	−0.0064 (10)	−0.0017 (9)
C12	0.0339 (18)	0.0202 (16)	0.0345 (19)	−0.0016 (13)	0.0017 (14)	−0.0006 (14)
C13	0.0307 (18)	0.0221 (17)	0.041 (2)	0.0001 (13)	0.0043 (15)	0.0014 (14)
C14	0.0302 (18)	0.0221 (16)	0.0361 (19)	−0.0037 (13)	0.0059 (14)	0.0018 (14)
C15	0.0317 (18)	0.0193 (16)	0.0355 (19)	−0.0017 (13)	0.0063 (14)	0.0031 (13)
C16	0.0351 (18)	0.0187 (16)	0.0325 (18)	−0.0032 (13)	0.0092 (14)	0.0017 (13)
C17	0.0324 (18)	0.0211 (16)	0.0304 (18)	−0.0026 (13)	0.0071 (14)	0.0017 (13)
C18	0.0337 (18)	0.0216 (16)	0.0307 (18)	−0.0056 (13)	0.0078 (14)	−0.0019 (14)
C19	0.0339 (18)	0.0199 (16)	0.0326 (18)	−0.0067 (13)	0.0061 (14)	0.0001 (13)
C20	0.0356 (18)	0.0214 (16)	0.0261 (17)	−0.0065 (13)	0.0060 (14)	−0.0011 (13)
C21	0.0349 (19)	0.0244 (17)	0.0321 (18)	−0.0067 (13)	0.0018 (15)	−0.0015 (14)
O21	0.0407 (13)	0.0284 (12)	0.0299 (12)	−0.0113 (10)	−0.0041 (10)	0.0033 (10)

### Geometric parameters (Å, º)

**Table:**

C1—C2	1.427 (4)	O11—H11	0.76 (4)
C1—C5	1.432 (4)	C12—C13	1.534 (4)
C1—C11	1.501 (4)	C12—H12A	0.9900
C1—Fe1	2.040 (3)	C12—H12B	0.9900
C2—C3	1.413 (4)	C13—C14	1.515 (4)
C2—Fe1	2.041 (3)	C13—H13A	0.9900
C2—H2	0.9500	C13—H13B	0.9900
C3—C4	1.423 (4)	C14—C15	1.521 (4)
C3—Fe1	2.043 (3)	C14—H14A	0.9900
C3—H3	0.9500	C14—H14B	0.9900
C4—C5	1.413 (4)	C15—C16	1.517 (4)
C4—Fe1	2.042 (3)	C15—H15A	0.9900
C4—H4	0.9500	C15—H15B	0.9900
C5—Fe1	2.038 (3)	C16—C17	1.523 (4)
C5—H5	0.9500	C16—H16A	0.9900
Fe1—C9	2.033 (3)	C16—H16B	0.9900
Fe1—C10	2.041 (3)	C17—C18	1.520 (4)
Fe1—C6	2.049 (3)	C17—H17A	0.9900
Fe1—C8	2.052 (3)	C17—H17B	0.9900
Fe1—C7	2.053 (3)	C18—C19	1.519 (4)
C6—C7	1.415 (4)	C18—H18A	0.9900
C6—C10	1.422 (4)	C18—H18B	0.9900
C6—H6	0.9500	C19—C20	1.518 (4)
C7—C8	1.420 (4)	C19—H19A	0.9900
C7—H7	0.9500	C19—H19B	0.9900
C8—C9	1.402 (5)	C20—C21	1.504 (4)
C8—H8	0.9500	C20—H20A	0.9900
C9—C10	1.410 (5)	C20—H20B	0.9900
C9—H9	0.9500	C21—O21	1.427 (3)
C10—H10	0.9500	C21—H21A	0.9900
C11—O11	1.439 (3)	C21—H21B	0.9900
C11—C12	1.512 (4)	O21—H21	0.83 (4)
C11—H11A	1.0000		
			
C2—C1—C5	107.1 (3)	C9—C8—H8	126.0
C2—C1—C11	127.5 (3)	C7—C8—H8	126.0
C5—C1—C11	125.4 (3)	Fe1—C8—H8	126.5
C2—C1—Fe1	69.60 (16)	C8—C9—C10	108.9 (3)
C5—C1—Fe1	69.37 (16)	C8—C9—Fe1	70.63 (18)
C11—C1—Fe1	127.9 (2)	C10—C9—Fe1	70.03 (18)
C3—C2—C1	108.7 (3)	C8—C9—H9	125.5
C3—C2—Fe1	69.85 (17)	C10—C9—H9	125.5
C1—C2—Fe1	69.47 (16)	Fe1—C9—H9	125.4
C3—C2—H2	125.7	C9—C10—C6	107.2 (3)
C1—C2—H2	125.7	C9—C10—Fe1	69.47 (18)
Fe1—C2—H2	126.6	C6—C10—Fe1	69.96 (17)
C2—C3—C4	107.8 (3)	C9—C10—H10	126.4
C2—C3—Fe1	69.69 (17)	C6—C10—H10	126.4
C4—C3—Fe1	69.54 (16)	Fe1—C10—H10	125.8
C2—C3—H3	126.1	O11—C11—C1	110.8 (2)
C4—C3—H3	126.1	O11—C11—C12	106.2 (2)
Fe1—C3—H3	126.2	C1—C11—C12	113.0 (2)
C5—C4—C3	108.3 (3)	O11—C11—C21	79.36 (14)
C5—C4—Fe1	69.60 (16)	C1—C11—C21	149.62 (17)
C3—C4—Fe1	69.67 (16)	O11—C11—H11A	108.9
C5—C4—H4	125.8	C1—C11—H11A	108.9
C3—C4—H4	125.8	C12—C11—H11A	108.9
Fe1—C4—H4	126.5	C21—C11—H11A	93.4
C4—C5—C1	108.1 (3)	C11—O11—H11	109.5
C4—C5—Fe1	69.87 (16)	C11—C12—C13	113.0 (3)
C1—C5—Fe1	69.49 (16)	C11—C12—H12A	109.0
C4—C5—H5	125.9	C13—C12—H12A	109.0
C1—C5—H5	125.9	C11—C12—H12B	109.0
Fe1—C5—H5	126.3	C13—C12—H12B	109.0
C9—Fe1—C5	120.61 (12)	H12A—C12—H12B	107.8
C9—Fe1—C1	156.53 (13)	C14—C13—C12	114.8 (3)
C5—Fe1—C1	41.13 (11)	C14—C13—H13A	108.6
C9—Fe1—C10	40.51 (13)	C12—C13—H13A	108.6
C5—Fe1—C10	106.43 (12)	C14—C13—H13B	108.6
C1—Fe1—C10	121.14 (13)	C12—C13—H13B	108.6
C9—Fe1—C2	160.79 (14)	H13A—C13—H13B	107.6
C5—Fe1—C2	68.63 (12)	C13—C14—C15	112.4 (2)
C1—Fe1—C2	40.93 (11)	C13—C14—H14A	109.1
C10—Fe1—C2	157.79 (13)	C15—C14—H14A	109.1
C9—Fe1—C4	106.89 (12)	C13—C14—H14B	109.1
C5—Fe1—C4	40.53 (11)	C15—C14—H14B	109.1
C1—Fe1—C4	68.75 (11)	H14A—C14—H14B	107.9
C10—Fe1—C4	122.90 (12)	C16—C15—C14	114.9 (2)
C2—Fe1—C4	68.28 (12)	C16—C15—H15A	108.5
C9—Fe1—C3	123.86 (13)	C14—C15—H15A	108.5
C5—Fe1—C3	68.59 (12)	C16—C15—H15B	108.5
C1—Fe1—C3	68.80 (12)	C14—C15—H15B	108.5
C10—Fe1—C3	159.82 (13)	H15A—C15—H15B	107.5
C2—Fe1—C3	40.46 (12)	C15—C16—C17	113.8 (2)
C4—Fe1—C3	40.79 (12)	C15—C16—H16A	108.8
C9—Fe1—C6	67.91 (12)	C17—C16—H16A	108.8
C5—Fe1—C6	124.07 (12)	C15—C16—H16B	108.8
C1—Fe1—C6	107.89 (12)	C17—C16—H16B	108.8
C10—Fe1—C6	40.69 (12)	H16A—C16—H16B	107.7
C2—Fe1—C6	122.93 (12)	C18—C17—C16	114.3 (2)
C4—Fe1—C6	160.03 (13)	C18—C17—H17A	108.7
C3—Fe1—C6	158.10 (12)	C16—C17—H17A	108.7
C9—Fe1—C8	40.16 (13)	C18—C17—H17B	108.7
C5—Fe1—C8	156.13 (12)	C16—C17—H17B	108.7
C1—Fe1—C8	161.73 (12)	H17A—C17—H17B	107.6
C10—Fe1—C8	68.02 (13)	C19—C18—C17	113.9 (2)
C2—Fe1—C8	125.22 (13)	C19—C18—H18A	108.8
C4—Fe1—C8	121.53 (12)	C17—C18—H18A	108.8
C3—Fe1—C8	108.17 (13)	C19—C18—H18B	108.8
C6—Fe1—C8	67.80 (13)	C17—C18—H18B	108.8
C9—Fe1—C7	67.93 (12)	H18A—C18—H18B	107.7
C5—Fe1—C7	161.19 (12)	C20—C19—C18	113.7 (2)
C1—Fe1—C7	124.82 (12)	C20—C19—H19A	108.8
C10—Fe1—C7	68.32 (12)	C18—C19—H19A	108.8
C2—Fe1—C7	109.00 (12)	C20—C19—H19B	108.8
C4—Fe1—C7	157.52 (13)	C18—C19—H19B	108.8
C3—Fe1—C7	122.55 (12)	H19A—C19—H19B	107.7
C6—Fe1—C7	40.36 (12)	C21—C20—C19	112.8 (2)
C8—Fe1—C7	40.47 (12)	C21—C20—H20A	109.0
C7—C6—C10	108.3 (3)	C19—C20—H20A	109.0
C7—C6—Fe1	69.98 (16)	C21—C20—H20B	109.0
C10—C6—Fe1	69.35 (17)	C19—C20—H20B	109.0
C7—C6—H6	125.9	H20A—C20—H20B	107.8
C10—C6—H6	125.9	O21—C21—C20	113.8 (2)
Fe1—C6—H6	126.4	O21—C21—C11	148.76 (17)
C6—C7—C8	107.6 (3)	O21—C21—H21A	108.8
C6—C7—Fe1	69.66 (17)	C20—C21—H21A	108.8
C8—C7—Fe1	69.73 (17)	C11—C21—H21A	91.3
C6—C7—H7	126.2	O21—C21—H21B	108.8
C8—C7—H7	126.2	C20—C21—H21B	108.8
Fe1—C7—H7	126.0	C11—C21—H21B	86.3
C9—C8—C7	108.0 (3)	H21A—C21—H21B	107.7
C9—C8—Fe1	69.21 (18)	C21—O21—H21	109.5
C7—C8—Fe1	69.81 (17)		

### Hydrogen-bond geometry (Å, º)

**Table:**

*D*—H···*A*	*D*—H	H···*A*	*D*···*A*	*D*—H···*A*
O11—H11···O21^i^	0.76	2.06	2.755 (3)	152
O21—H21···O11^ii^	0.83	1.90	2.726 (3)	175
C6—H6···O21^i^	0.95	2.60	3.380 (4)	140

Symmetry codes: (i) −*x*+1/2, *y*−1/2, −*z*+3/2; (ii) −*x*+1/2, −*y*+3/2, −*z*+2.
